# Anterior approach and robotic assistance associated with shorter operative times and lower transfusion requirements in conversion total hip arthroplasty

**DOI:** 10.1007/s00590-026-04765-3

**Published:** 2026-05-06

**Authors:** Ryan Riley, Ellen Lutnick, Nicholas Frappa, Jacob Geiger, Amelia Simpson, Aaron Leininger, Evgeny Dyskin

**Affiliations:** 1 Department of Orthopaedic Surgery, University at Buffalo, State University of New York, Buffalo, USA; 2 Department of Orthopedic Surgery, Yale University, New Haven, USA

**Keywords:** Direct anterior approach, Posterior approach, Total hip arthroplasty, THA, Hip surgery conversion, Revision total hip arthroplasty

## Abstract

**Background:**

This study aims to describe a cohort of patients who underwent conversion to total hip arthroplasty (THA), comparing surgical approach and robotic assistance.

**Methods:**

Patients ≥ 18 years who underwent conversion to THA between January 1, 2015 and May 6, 2024, were retrospectively reviewed, and additionally contacted via telephone for updated patient reported outcome measures (PROMs), including the Hip Disability and Osteoarthritis Outcome Score, Joint Replacement (HOOS, JR) questionnaire. Surgical approach and robotic assistance were per surgeon preference, with consideration for the approach of the previous hip surgery. Descriptive statistics were calculated, and continuous variables analyzed using the Student’s T-Test, the Wilcoxon Ranked Sum Test. Categorical variables were compared using Chi-Squared Tests and Fisher’s Exact Tests, and outcomes analyzed with logistic regression in R statistical software.

**Results:**

83 patients met inclusion (53% anterior approach; 26.5% robotic assistance). Conversion via anterior approach demonstrated shorter operative times than posterior (by 45.9 min; *p* < 0.001), in addition to fewer postoperative transfusions (22.7% vs. 53.8%; *p* = 0.006). Length of stay (LOS), and fluoroscopy time were not significant. When stratified by previous surgery 37 patients were converted from prior intramedullary nail, 16 via the anterior approach. Mean operative time remained shorter (*p* = 0.003) and transfusion remained less (*p* = 0.015) compared to posterior conversion in this subset. Robotic assistance was associated with shorter operative times (by 44.9 min *p* < 0.001), and shorter LOS (*p* < 0.001). Zero robotic-assisted patients required transfusion within 24 h postoperatively compared to 50.8% of non-robotic cases (*p* < 0.001), although transfusion risk was not significant considering approach or robotic assistance after multivariable regression controlling for preoperative hemoglobin. PROMs were equivocal between compared groups (mean HOOS Jr 11.0 ± 5.1, response rate 42.2%).

**Conclusion:**

Conversion THA demonstrates acceptable outcomes, with documented overall improvements in postoperative function. The anterior approach and robotic assistance were associated with significantly shorter surgical times, and less risk of transfusion, potentially demonstrating some advantage in this conversion scenario, although patient selection may have also influenced outcomes.

## Introduction

Total hip arthroplasty (THA) is one of the most successful orthopaedic procedures, with utilization projected to increase substantially. Consequently, by 2030, revision total hip arthroplasty (THA) is projected to increase by over 40% [1], a trend likely driven by increased life expectancy, expanded indications, and rising rates of primary THA in younger patients. One important contributor to this trend is the growing frequency of conversion THA following failed fixation of proximal femoral fractures, especially those managed with cephalomedullary nails or other intramedullary implants [2–4]. These procedures are technically demanding and carry a higher risk of complications than primary THA [5, 6]. Previous series have reported longer operative times, greater blood loss, and increased rates of dislocation, infection, or trochanteric nonunion in conversion THA cases [7, 8].

Surgical approach in conversion THA plays a critical role in exposure, complication avoidance, and recovery. Though traditionally described for primary THA, the anterior approach has gained attention for its potential benefits in conversion scenarios [9–11], allowing for supine positioning, intraoperative fluoroscopy, and preservation of posterior soft tissues, which may enhance stability and enable more precise implant positioning even in cases requiring hardware removal or complex reconstruction [10]. Robotic-assisted THA has additionally emerged as a promising advancement, offering improved component alignment and surgical reproducibility [12–14]. While initially applied in the setting of primary THA, robotic systems have increasingly been explored in complex surgical scenarios, including revision arthroplasty with acetabular reconstruction [15] and conversion THA following hardware removal [16]. These settings may particularly benefit from the enhanced precision of robotics due to altered anatomy and constrained working spaces, although the effect on patient functional outcomes remains mixed.

Despite the existing literature surrounding conversion THA, there exists limited comparative data related to surgical approach and use of robotic assistance. This study aims to (1) describe a single-surgeon cohort of patients who underwent conversion to THA, and (2) compare surgical metrics and outcomes between anterior versus posterior approaches, and use of robotic-assisted versus non-robotic techniques.

## Methods

Retrospective chart review was performed for adult patients ≥ 18 years who underwent conversion THA by one arthroplasty fellowship trained surgeon between January 1, 2015-May 6, 2024, representing the period from the start of his practice performing conversion THA via various surgical approaches. Patients were initially identified by Current Procedural Terminology code 27,132 for conversion to hip arthroplasty, and charts screened by a trained research staff member and an orthopaedic resident. Patient demographics, injury, and treatment data were recorded, including types of primary surgery, the approach utilized for conversion (i.e. anterior based via the direct anterior (DA) or anterolateral approach versus posterior via the Kocher-Langenbeck approach) and use of robotic assistance.

Patients were selected for surgical approach and use of robotic assistance per surgeon preference, with consideration for the surgical approach utilized for the previous hip surgery. All conversion procedures were performed in a single setting, with removal of any previous instrumentation and conversion to THA under one episode of anesthesia. For DA approach, the Smith Peterson approach with Heuter interval modification was utilized, and previous incisions considered as accessory portals to insert straight guides or reamers, or portals as described by Keggi [17]. With difficult anatomy, fluoroscopy helped localize appropriate incision placement. Postoperatively, patients were typically made to be weight bearing as tolerated with assistive device as needed, and anterior or posterior hip precautions were followed depending on the approach utilized for the conversion. Patients were admitted for inpatient stay and discharged when deemed fit by physical therapy evaluation.

Patients who met inclusion were additionally contacted via telephone by trained research staff members for an updated survey of patient reported outcome measures (PROMs), including the Hip Disability and Osteoarthritis Outcome Score, Joint Replacement (HOOS, JR) questionnaire, a six-item survey, with score range from 0 to 30, and higher scores indicating worse function [18]. Patient verbal consent was obtained and documented prior to survey administration.

### Data analysis

Continuous variables were summarized as mean ± standard deviation, and categorical variables as counts and percentages. Group comparisons for continuous outcomes were performed using Welch’s t-tests when variance equality was not assumed or Wilcoxon rank-sum tests for non-normally distributed data. Categorical variables were compared using Fisher’s exact tests. Normality was evaluated visually and by Shapiro–Wilk testing. Correlations between continuous variables were assessed using Spearman’s rank coefficients.

Multivariable models included covariates selected based on clinical relevance and prior univariable significance thresholds. Because procedures were performed across multiple years, year of surgery was included as a covariate in multivariable models evaluating operative time, transfusion, and length of stay to account for potential temporal or learning-curve effects. Firth-penalized logistic regression was used for binary outcomes with rare events to reduce small-sample bias and handle separation. Linear regression was used for continuous outcomes (e.g., operative time, PROM total), and logistic regression for dichotomous outcomes (e.g., transfusion, hip pain, composite complications).

Multiple-comparison correction for item-level PROM analyses was performed using the Benjamini–Hochberg false-discovery rate procedure. Statistical significance was defined as a two-sided *p* < 0.05. All analyses were conducted in R version 4.5.0 (R Foundation for Statistical Computing, Vienna, Austria) using the packages tidyverse, ggplot2, rstatix, logistf, effsize, broom, and psych.

This study was Institutional Review Board approved. All data was collected, and descriptive statistics were generated using REDCap (Research Electronic Data Capture), a secure, web-based software platform designed to support data capture for research studies [19, 20]. Patient consent was obtained verbally via telephone and electronically recorded in REDCap. Data management complied with HIPAA regulations, and the research team were trained in data security and patient confidentiality.

## Results

### Baseline characteristics

Among 83 patients who underwent conversion THA, prior index procedures included intramedullary nailing (IMN) in 37 patients (44.6%), open reduction–internal fixation (ORIF) in 27 (32.5%), closed reduction–percutaneous pinning (CRPP) in 10 (12.0%), osteotomy in 5 (6.0%), hemiarthroplasty in 3 (3.6%), and Girdlestone resection in 1 (1.2%). The conversion THA approach was anterior based in 44 patients (53.0%) and posterior in 39 (47.0%). Robotic assistance was used in 22 of 83 cases (26.5%), including 15 of 44 anterior procedures (34.1%) and 7 of 39 posterior procedures (17.9%).

Robotic-assisted procedures were introduced in 2018, and both robotic and non-robotic procedures were performed during overlapping years throughout the study period. Compared with non-robotic cases, robotic-assisted patients were younger and had lower prevalences of hypertension and diabetes (Table 1). However, when stratified by surgical approach, baseline comorbidity profiles were similar between anterior and posterior cohorts (all *p* > 0.05; Table 2).

**Table 1:** Patient demographics by group

Characteristic	Overall *N* = 83	No robot *N* = 61	Robot *N* = 22	*p* value
*Approach*				0.135
Anterior	44 (53%)	29 (48%)	15 (68%)	
Posterior	39 (47%)	32 (52%)	7 (32%)	
*Gender*				1.000
Male	38 (46%)	28 (46%)	10 (45%)	
Female	45 (54%)	33 (56%)	12 (55%)	
*Race*				0.475
White/Caucasian	51 (84%)	34 (79%)	17 (94%)	
Black or African American	9 (11%)	8 (19%)	1 (6%)	
*Comorbidities*				
Cardiovascular Disease	34 (41%)	27 (44%)	7 (32%)	0.449
Hypertension	52 (63%)	44 (73%)	8 (36%)	0.005
Hyperlipidemia	34 (41%)	28 (47%)	6 (27%)	0.140
COPD	10 (12%)	7 (12%)	3 (14%)	0.720
Asthma	13 (16%)	9 (15%)	4 (18%)	0.738
Cancer	6 (7%)	5 (8%)	1 (5%)	1.000
CKD	12 (15%)	11 (18%)	1 (5%)	0.168
Diabetes	17 (21%)	16 (27%)	1 (5%)	0.033
Cerebrovascular Disease	9 (11%)	7 (12%)	2 (9%)	1.000
Age (years)	62 ± 17	65 ± 16	53 ± 18	0.017
BMI (kg/m^2^)	30 ± 8	30 ± 8	30 ± 7	0.993
Length of Follow-up (Days)	148 (45–376)	174 (85–533)	92 (67–318)	0.172

^a^n (%); Mean ± SD; Median (IQR)

^b^Categorical variables were compared using Fisher’s exact tests. Continuous variables were compared using Welch’s two-sample t-tests or Wilcoxon rank-sum tests where appropriate.

**Table 2:** Patient demographics by group

Characteristic	Overall *N* = 83	Anterior *N* = 44	Posterior *N* = 39	*p* value
*Robot versus no robot*				0.135
No Robot	61 (73%)	29 (66%)	32 (82%)	
Robot	22 (27%)	15 (34%)	7 (18%)	
*Gender*				0.123
Male	38 (46%)	24 (55%)	14 (36%)	
Female	45 (54%)	20 (45%)	25 (64%)	
*Race*				0.531
White/Caucasian	51 (84%)	33 (85%)	18 (82%)	
Black or African American	9 (15%)	6 (15%)	3 (14%)	
*Comorbidities*				
Cardiovascular disease	34 (41%)	19 (43%)	15 (38%)	0.823
Hypertension	52 (63%)	28 (64%)	24 (62%)	1.000
Hyperlipidemia	34 (41%)	16 (36%)	18 (46%)	0.382
COPD	10 (12%)	6 (14%)	4 (10%)	0.743
Asthma	13 (16%)	8 (18%)	5 (13%)	0.558
Cancer	6 (7%)	3 (7%)	3 (8%)	1.000
CKD	12 (14%)	7 (16%)	5 (13%)	0.762
Diabetes	17 (20%)	7 (16%)	10 (26%)	0.292
Cerebrovascular disease	9 (11%)	4 (9%)	5 (13%)	0.728
Age (years)	62 ± 17	60 ± 19	63 ± 17	0.529
BMI (kg/m^2^)	30 ± 8	30 ± 8	31 ± 7	0.779
Length of Follow-up (Days)	148 (45–376)	118 (69–392)	168 (90–533)	0.330

^a^Values are presented as n (%) for categorical variables and mean ± SD or Median (IQR) for continuous variables

^b^Categorical variables were compared using Fisher’s exact tests; continuous variables were compared using Welch two-sample t-tests or Wilcoxon rank-sum tests where appropriate

### Operative metrics

Across the 83 conversions to total hip arthroplasty, mean operative time was 180 ± 57 min and estimated blood loss (EBL) averaged 721 ± 564 milliliters (mL). When stratified by approach, anterior procedures demonstrated significantly shorter operative times than posterior conversions (158 ± 57 vs. 204 ± 48 min; *p* < 0.001). Mean EBL was lower for anterior cases (653 ± 454 mL) than posterior (797 ± 664 mL; *p* = 0.26), and preoperative hemoglobin (Hb) was higher in the anterior cohort (13.4 ± 1.8 vs. 11.8 ± 1.9 g/dL; *p* < 0.001). Postoperative transfusion within 24 h of surgery was significantly lower amongst those patients treated with an anterior approach (22.7% vs. 53.8%; *p* = 0.006). However, in multivariable Firth logistic regression adjusting for age, hypertension, diabetes, chronic kidney disease, surgical approach, and preoperative hemoglobin, surgical approach was not independently associated with transfusion (OR 2.66, 95% CI 0.75–9.87; *p* = 0.13). Lower preoperative hemoglobin demonstrated a trend toward increased transfusion risk (OR 0.72 per g/dL increase; *p* = 0.059). Although the anterior group experienced a significantly greater decrease in Hb from baseline (− 2.9 ± 1.6 vs. − 2.2 ± 1.4 g/dL; *p* = 0.04), their absolute postoperative Hb remained significantly higher than the posterior group (10.5 ± 1.8 vs. 9.6 ± 1.7 g/dL; *p* = 0.03). Length of stay (LOS; 3.6 ± 4.1 vs. 4.7 ± 3.5 days; *p* = 0.21) was not significantly different. Fluoroscopy time demonstrated a zero-inflated distribution with many procedures performed without fluoroscopy; median fluoroscopy time was 0 s in both anterior and posterior approaches (range 0–61.4 vs. 0–51.6 s; Wilcoxon *p* = 0.52). Across approaches, there was no statistically significant difference in overall complication rate 14/44 (31.8%) vs. 12/39 (30.8%) (*p* = 1.00) and no differences for any individual complication subtype (all *p* > 0.05; Table 3).

**Table 3:** Outcomes—all anterior versus posterior approach

Summary	Anterior	Posterior	*p*-value
N	44	39	
*Surgical metrics*			
Operative time (min)	158.0 ± 57	203.9 ± 48	< 0.001
Estimated blood loss (mL)	653.4 ± 454	797 ± 664	0.26
Fluoroscopy time (sec)	0 (0–61.4)	0 (0–51.6)	0.52
Length of stay (days)	3.6 ± 4.1	4.7 ± 3.5	0.21
*Hemoglobin*			
Pre-op hemoglobin (g/dL)	13.4 ± 1.8	11.8 ± 1.9	< 0.001
Post-op hemoglobin (g/dL)	10.5 ± 1.8	9.6 ± 1.7	0.03
Δ Hemoglobin (post − pre) (g/dL)	-2.9 ± 1.6	-2.2 ± 1.4	0.04
*PROMs*			
PROM total (0–30)	10.1	13.1	0.25
Hip pain	10/25 (40.0%)	5/10 (50.0%)	0.71
Transfusion within 24 h	10/44 (22.7%)	21/39 (53.8%)	0.006
*Complications*			
Total	14/44 (31.8%)	12/39 (30.8%)	1.0
Infection	3/44 (6.8%)	2/39 (5.1%)	1.0
Dislocation	4/44 (9.1%) (Occurred postoperatively at 1 week, 1 month, 2 months and 3 months)	2/39 (5.1%) (Occurred postoperatively 1 month and 2 months)	0.68
Hematoma	1 (2.3%)	1 (2.6%)	1.0
Fracture/periprosthetic fracture	4 (9.1%)	2 (5.1%)	0.68
Other	1 Postoperative Bleeding 1 Hypotension	1 Greater Trochanter Bursitis 1 DVT 1 Acute blood loss anemia 1 Revision for instability 1 Skin Ulcer	0.25

Data are presented as mean ± standard deviation, median (range), or n (%), as appropriate. P-values were calculated using Student’s t-test or the Wilcoxon rank-sum test for continuous variables and Pearson’s chi-square or Fisher’s exact test for categorical variables. Fluoroscopy time demonstrated a zero-inflated distribution and is therefore reported as median (range). Complication rates are calculated based on the total cohort size (N). PROM percentages are calculated based on the number of patients with completed data

Upon isolation of the subset of 37 patients converted from IMN, 16 underwent conversion via anterior and 21 via posterior approach. Operative time remained shorter in the anterior group (171 ± 42 vs. 222 ± 53 min; *p* = 0.003), with numerically lower EBL (769 ± 499 vs. 1086 ± 789 mL; *p* = 0.15). Pre- and postoperative Hb were higher in the anterior cohort (13.2 ± 1.6 vs. 11.1 ± 1.4 g/dL, *p* = 0.0003; 10.2 ± 1.9 vs. 8.8 ± 1.4 g/dL, *p* = 0.019), whereas Hb change, LOS, and fluoroscopy time were similar between groups. Transfusion occurred less often with the anterior approach (37.5% vs. 81.0%; *p* = 0.015), and infection and overall complication rates did not differ (both *p* = 1.00).

Robotic-assisted procedures (*n* = 22) had markedly shorter operative times (147 ± 45 vs. 191 ± 57 min; *p* < 0.001) and lower EBL (500 ± 209 vs. 786.3 ± 648 mL; *p* = 0.002) than non-robotic cases (*n* = 61). Pre-operative Hb was higher among robotic patients (13.8 ± 1.7 vs. 12.3 ± 2.0 g/dL; *p* = 0.003), while postoperative Hb and ΔHb were similar. LOS was substantially shorter in the robotic cohort (1.5 ± 0.7 vs. 5.1 ± 4.1 days; *p* < 0.001). Fluoroscopy time did not differ between robotic and non-robotic procedures (median 0 s in both groups; Wilcoxon *p* = 0.905). Transfusion within 24 h occurred in none of the robotic cases versus 31 of 61 (50.8%) non-robotic cases (*p* < 0.001). In multivariable Firth logistic regression adjusting for age, hypertension, diabetes, chronic kidney disease, surgical approach, preoperative hemoglobin, and year of surgery, robotic assistance demonstrated a trend toward lower odds of transfusion but did not reach statistical significance (*p* = 0.060). In multivariable linear regression models adjusting for age, hypertension, diabetes, chronic kidney disease, surgical approach, and year of surgery, robotic assistance remained independently associated with shorter operative time (β − 36.6 min, 95% CI − 65.0 to − 8.1; *p* = 0.013) and shorter length of stay (β − 3.31 days, 95% CI − 5.26 to − 1.37; *p* = 0.001). Year of surgery was not independently associated with operative time or length of stay. Sensitivity analyses restricted to years in which both robotic and non-robotic procedures were performed contemporaneously (2018–2023) demonstrated similar findings, with robotic assistance remaining associated with shorter operative time (β − 57.0 min, 95% CI − 94.7 to − 19.4; *p* = 0.004) and shorter length of stay (β − 2.24 days, 95% CI − 4.13 to − 0.34; *p* = 0.022). Infection (4.5% vs. 6.6%; *p* = 1.00) and overall complication rates (22.7% vs. 34.4%; *p* = 0.42) were comparable (Table 4).

**Table 4:** Outcomes—robotic versus no-robotic assistance

Summary	No robot	Robot	*p*-value
N	61	22	–
*Surgical metrics*			
Operative time (min)	191.5 ± 57	146.6 ± 45	< 0.001
Estimated blood loss (mL)	786.3 ± 647.8	500.0 ± 208.7	0.002
Fluoroscopy time (sec)	0 (0–51.6)	0 (0–61.4)	0.905
Length of stay (days)	5.1 ± 4.0	1.5 ± 0.7	< 0.001
*Hemoglobin*			
Pre-op hemoglobin (g/dL)	12.3 ± 2.0	13.8 ± 1.7	0.003
Post-op hemoglobin (g/dL)	9.8 ± 1.8	10.7 ± 1.6	0.639
Δ Hemoglobin (post − pre) (g/dL)	-2.5 ± 1.6	-3.1 ± 1.5	0.14
*PROMs*			
PROM total (0–30)	11.6	9.4	0.34
Hip pain	10/25 (40.0%)	5/10 (50.0%)	0.71
Transfusion within 24 h	31/61 (50.8%)	0/22 (0.0%)	< 0.001
*Complications*			
Total	21/61 (34.4%)	5/22 (22.7%)	0.42
Infection	4/61 (6.6%)	1/22 (4.5%)	1.0
Dislocation	5/61 (8.2%) (Occurred at 1 week, 1 month, 2 months, 2 months, and 3 months)	1/22 (4.5%) (Occurred postoperatively at 1 month)	1.0
Hematoma	2/61 (3.3%)	0	1.0
Fracture/Periprosthetic Fracture	4/61 (6.6%)	2/22 (9.1%)	0.65
Other	1 Greater Trochanteric Bursitis 1 Deep Vein Thrombosis (DVT) 1 Acute Blood Loss Anemia 1 Postoperative Bleeding 1 Hypotension 1 Skin Ulcer	1 Revision for instability	0.67

Data are presented as mean ± standard deviation, median (range), or n (%), as appropriate. P-values were calculated using Student’s t-test or the Wilcoxon rank-sum test for continuous variables and Pearson’s chi-square or Fisher’s exact test for categorical variables. Fluoroscopy time demonstrated a zero-inflated distribution and is therefore reported as median (range). Complication rates are calculated based on the total cohort size (N). PROM percentages are calculated based on the number of patients with completed data

## Perioperative functional outcomes

Functional independence followed similar patterns. Before surgery, independent ambulation was observed in 49% of anterior versus 41% of posterior patients (*p* = 0.51); at six months, 67% versus 52% (*p* = 0.29); and at one year, 88% versus 63% (*p* = 0.35; Table 5). Robotic patients were more frequently independent both preoperatively (86% vs. 32%; *p* < 0.001) and at six months (91% vs. 51%; *p* = 0.019). At one year, all evaluable robotic patients (5/5) and 63% (12/19) of non-robotic patients were independent (*p* = 0.27; Table 6).

**Table 5:** Postoperative ambulation by surgical approach

Characteristic	Ambulation status	Overall *N* = 83	Anterior *N* = 44	Posterior *N* = 39	*P* value
*Preoperative ambulation*					
Independent		37 (46%)	21 (49%)	16 (41%)	0.51
Assistance		44 (54%)	22 (51%)	23 (59%)	
*6-Month follow-up ambulation*					
Independent		33 (59%)	18 (67%)	15 (52%)	0.29
Assistance		23 (41%)	9 (33%)	14 (48%)	
*1-Year follow-up ambulation*					
Independent		17 (71%)	7 (88%)	10 (63%)	0.35
Assistance		7 (29%)	1 (12%)	6 (38%)	

Data presented as n (%). Percentages are calculated based on the number of patients with available follow-up data at each time point, excluding missing values. P-values were calculated using Pearson’s Chi-squared test or Fisher’s Exact test where expected cell counts were < 5

**Table 6:** Postoperative ambulation by robotic assistance

Characteristic	Overall *N* = 83	Non-robotic *N* = 61	Robotic *N* = 22	*P* value
*Preoperative ambulation*				
Independent	37 (46%)	19 (32%)	18 (86%)	< 0.001
Assistance	44 (54%)	41 (68%)	3 (14%)	
*6-Month follow-up ambulation*				
Independent	33 (59%)	23 (51%)	10 (91%)	0.019
Assistance	23 (41%)	22 (49%)	1 (9%)	
*1-Year follow-up ambulation*				
Independent	17 (71%)	12 (63%)	5 (100%)	0.27
Assistance	7 (29%)	7 (37%)	0 (0%)	

Data presented as n (%). Percentages are calculated based on the number of patients with available follow-up data at each time point, excluding missing values. P-values were calculated using Pearson’s Chi-squared test or Fisher’s Exact test where expected cell counts were < 5

### Patient-reported outcomes

Among 83 patients, 35 (42.2%) completed postoperative PROMs with a median follow-up of 148 days (IQR 331). 15 of these (42.9%) reported residual hip pain. The mean total HOOS Jr score was 11.0 ± 5.1. Responses suggested generally mild activity limitations across domains: approximately two-thirds of patients reported none or only mild difficulty with bending, and over three-quarters reported minimal difficulty lying down. Severe or extreme difficulty was uncommon across all items (< 10% for any domain).

When stratified by robotic assistance, robotic versus non-robotic cases showed similar hip-pain prevalence (50% vs. 40%; *p* = 0.71) and total scores (9.4 vs. 11.6; Welch *p* = 0.16; Wilcoxon *p* = 0.34), with no item-level differences after Benjamini–Hochberg adjustment (all *p* ≥ 0.44). By surgical approach, anterior and posterior groups had comparable hip-pain rates (40% vs. 50%; *p* = 0.71) and total scores (10.1 vs. 13.1; Welch *p* = 0.25; Wilcoxon *p* = 0.35). Within the IMN subset, anterior and posterior conversions demonstrated similar findings (hip pain 44.4% vs. 66.7%, *p* = 0.61; total 10.2 vs. 14.8, Welch *p* = 0.25; Wilcoxon *p* = 0.29). No comparisons reached statistical significance, though small-to-moderate effect sizes favored robotic and anterior approaches.

Across PROM completers, individual comorbidities were not associated with PROM totals or hip pain after multiple-comparison adjustment (all adjusted *p* ≥ 0.22). Asthma showed a nominal association with pain (100% vs. 35%), which did not persist after correction (q = 0.24). Comorbidity burden (count) did not correlate with PROM total (ρ = − 0.02; *p* = 0.93) and was similar across 0–1, 2–3, and ≥ 4 comorbidities (*p* = 0.99). Firth logistic regression showed no association between comorbidity burden and hip pain, whether modeled continuously (OR 1.10 per comorbidity; 95% CI 0.73–1.69; *p* = 0.64) or categorically (≥ 4 vs. 0–1; *p* = 0.59). Sensitivity analyses adjusting for age and approach produced similar results (PROM β = − 0.17; *p* = 0.79; pain OR 1.09; *p* = 0.72).

To evaluate potential nonresponse bias, baseline characteristics of PROM responders and non-responders were compared. Responders and non-responders did not differ significantly in age (61.7 vs. 61.6 years; *p* = 0.98), hypertension (72.0% vs. 58.6%; *p* = 0.32), diabetes (16.0% vs. 22.4%; *p* = 0.57), or return-to-operating-room rates (12.0% vs. 14.5%; *p* = 1.00).

## Discussion

Conversion of failed femoral fracture fixation to total hip arthroplasty (THA) is a complex but increasingly necessary procedure given the aging population and the rising incidence of fragility fractures including hip fractures. This retrospective cohort specifically examined the outcomes of conversion THA via anterior vs. posterior approaches, as well as conversion via robotic assistance, comparisons which remains underrepresented in the literature. This analysis revealed that the anterior approach and the use of robotic assistance can be safely and effectively employed for single-stage conversion THA, with outcomes that compare favorably to those achieved via alternative approaches. Of this cohort, a specific subset converted after prior IMN demonstrated similar results favoring anterior approach, highlighted in isolation given the technical challenge associated with this conversion procedure.

Amongst the presented results, anterior based approaches offered significantly shorter surgical times when compared directly to posterior, which remained true considering subset analysis of conversion from prior IMN. This is a significant finding that has not been previously reported in the literature. Additionally, anterior-based approaches demonstrated numerically lower mean EBL when compared to posterior cases, though this did not reach statistical significance, while being associated with significantly lower postoperative transfusion requirements. Amongst those cases converted after prior IMN, the anterior approach was additionally associated with significantly fewer postoperative transfusion requirements. Consideration for a larger patient population may discover overall statistical significance between the anterior and posterior groups when blinding for index surgery. These findings could prove advantageous, as factors including time under anesthesia and blood loss are major concerns in the conversion scenario.

Complications of conversion surgery are inherently more frequent given the complexity compared to primary THA. These data demonstrated no statistical difference in the overall complication rate between the anterior and posterior approach (*p* = 1.0). This aligns with emerging reports supporting the safety and efficacy of the anterior approach in the setting of complex hip arthroplasty, including acetabular augmentation, subtrochanteric osteotomies, and femoral revisions through extensile exposures without compromising nerve integrity [21–23]. This cohort demonstrates comparable, if not favorable complication risk profiles compared to prior cohorts. Prior reported dislocation rates range from 5 to 22% in addition to highlighting risk of periprosthetic fractures during conversion THA via posterior approaches [2–4, 24]. Notably, Oinuma et al. [23] reported zero dislocations in a complex primary THA cohort using the DA approach in combination with subtrochanteric shortening osteotomy. Yun et al. [11] similarly reported no dislocations in their DA conversion series following failed femoral neck fixation, further supporting the stability advantages of the DA approach in challenging cases. Preservation of the posterior soft tissue structures likely contributes to this enhanced stability [21–23, 25, 26]. Additionally, use of intraoperative fluoroscopy in the supine position allows for precise component placement and verification of hardware removal, potentially reducing the risk of malposition-related instability [10, 23].

Considering functional outcomes, Brooks et al. conducted a cohort study of 23 patients undergoing conversion THA after acetabular fixation via DA approach, reporting no deep infections or neurovascular injuries, and only one dislocation (4.5%), resulting in an average Harris Hip Score of 92 at follow-up [9]. The functional outcomes represented by the current cohort were highly favorable, with over 70% of patients gaining independent ambulation after conversion, representing a 30% increase compared to preoperative ambulation status. This is consistent with prior literature demonstrating the relative improvements in mobility and quality of life after conversion THA in indicated patients [3, 8, 10]. Patient-reported outcomes in this series specifically represented via HOOS, JR scores were comparable to those reported in primary THA [1]; however, conclusions from this data are limited by the response rate (42.2%).

These results also highlight the potential for robotic assistance to improve efficiency and reduce transfusion rates in conversion THA. This study demonstrated no patients who underwent robotic assisted conversion required transfusion within 24 h post-op compared to approximately 50% without robotic assistance. While encouraging, this significant decrease in risk of transfusion may be a function of these patients being optimized candidates, including this cohort being younger than those treated without robotic assistance, rather than a result of the technology itself. Additionally, robotic-assisted conversions had markedly shorter operative times by over 40 min. In the primary arthroplasty setting, robotic assisted THA has been demonstrated to improve the accuracy of implant positioning, although this association with complication rates, implant survival, and patient functional outcomes is still debated [12–14, 27–29]. In the conversion setting, this reduction in operative time could potentially reflect more efficient hardware removal, or less complex anatomy/scarring; however, these presented results could also reflect ease of final implant positioning with the use of robotic assistance.

While the use of robotics in primary THA is well established, its application in complex conversion and revision scenarios is a promising frontier with growing interest. One limited case series described simultaneous hardware removal and conversion to THA with improvement in pain and quality of life at 1 year follow up in two patients [16]. A recent retrospective review of 54 patients examining robotic assisted THA in acetabular revision reported a 43-point increase in Harris Hip Score post-operatively, with an overall satisfaction rate of 86% and no radiological failures at 2-year follow up [15]. These reports reinforce that robotic assistance can be safely integrated into complex hip reconstructions, an assertion demonstrated in our own study where implants were successfully removed, and components were placed accurately in a single-stage procedure.

The present study contributes to the above growing body of literature, with finding robotic-assistance to be associated with significantly shorter length of stay and substantially lower transfusion within 24 h postoperatively in the complex conversion setting. Additionally, infection rates and complications were similar between robotic and non-robotic cohorts, demonstrating overall relative safety, as well as comparable prevalence of continued hip pain and PROM scores postoperatively. Despite these encouraging results, the literature related to robotic-assisted conversion THA remains limited, and this study contributes to this emerging field.

There are several limitations to our study. First, the retrospective design and single institution setting introduce potential selection bias, particularly in the decision to use robotic assistance. Additionally, the sample size, while representative of one of the largest comparative conversion THA series to date, remains modest; the follow-up period is additionally limited to early and midterm outcomes. Longer-term follow-up is necessary to assess implant survivorship and durability of functional gains. Finally, the surgeries were performed by a single surgeon, limiting generalizability. Despite these limitations, this study provides valuable insight into the potential benefits of anterior-based and robotic assisted approaches for conversion THA, found to be associated with enhanced surgical efficiency and minimized blood loss. Future multicenter studies with larger cohorts and randomized designs are warranted to confirm these findings and establish best practices for approach selection in conversion THA.

## Conclusion

Conversion THA is a technically challenging procedure, without true comparison described in the literature with regards to the efficacy and safety of the surgical approaches and technical options available. This analysis describes the overall efficacy of conversion THA by a variety of approaches highlighted in this cohort, with a relatively low complication risk profile, good functional outcomes, and PROMs comparative to primary hip arthroplasty. Specifically, this comparative analysis supports the efficacy and safety of anterior-based and robotic assisted approaches for conversion THA as associated with increased operative efficiency and less blood loss, although, after controlling for preoperative hemoglobin and other patient factors, transfusion risk was no longer significant, suggesting that patient selection also influenced outcomes. Future studies should examine other patient and surgical factors with the aim of further optimizing outcomes in these challenging cases.

## Data Availability

Data is available upon reasonable request to corresponding author.
